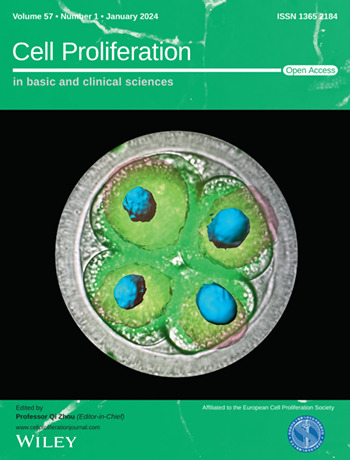# Featured Cover

**DOI:** 10.1111/cpr.13597

**Published:** 2024-01-06

**Authors:** Mohamed Aboul Ezz, Masashi Takahashi, Rocío Melissa Rivera, Ahmed Zaky Balboula

## Abstract

The cover image is based on the Original Article *Cathepsin L regulates oocyte meiosis and preimplantation embryo development* by Mohamed Aboul Ezz et al., https://doi.org/10.1111/cpr.13526.